# Assessment of the Efficacy of Low-Density Pedicle Screw Construction Correction of Adolescent Idiopathic Scoliosis: A Prospective Single-Center Study

**DOI:** 10.7759/cureus.48797

**Published:** 2023-11-14

**Authors:** Mahmoud Ebrahim Eladl, Mostafa Abdel-Samie Rabee, Ahmed Mohammed Taha

**Affiliations:** 1 Neurosurgery, Faculty of Medicine, Al-Azhar University, Damietta, EGY

**Keywords:** cobb angles, proximal junctional kyphosis, deformity correction, low-density pedicle screw fixation, adolescent idiopathic scoliosis

## Abstract

Background

Adolescent idiopathic scoliosis (AIS) poses physical and psychological challenges for affected individuals, necessitating effective and less invasive treatment approaches. This study aimed to evaluate the efficacy of low-density posterior pedicle screw fixation in AIS correction, exploring its impact on deformity parameters, patient quality of life, and potential complications.

Methodology

A prospective study involving 20 AIS patients, employing low-density pedicle screw fixation, was conducted. Clinical, radiological, and Scoliosis Research Society (SRS-22) outcomes were assessed. Data analysis was conducted using SPSS version 26 software (IBM Corp., Armonk, NY, USA).

Results

Significant reductions in major and minor curve Cobb angles were observed (mean major curve reduction: 79.14%, p < 0.001; mean minor curve reduction: 68.91%, p < 0.001), indicating substantial deformity correction. As measured by the SRS-22 questionnaire, quality of life showed noteworthy improvements (mean pain score increase: 0.54, p < 0.05; mean self-image score increase: 1.22, p < 0.01), reflecting enhanced patient satisfaction and well-being. Complications were documented in four (20%) cases, including infection, adding-on phenomenon, proximal junctional kyphosis, and thoracic hypokyphosis.

Conclusions

Our study highlights the efficacy of low-density pedicle screw constructs in AIS correction. Significant deformity reductions and improved quality of life underscore the success of the approach. However, long-term studies with larger cohorts are crucial for confirming durability.

## Introduction

Adolescent idiopathic scoliosis (AIS) is one of the most common spinal deformities affecting adolescents, with a prevalence of approximately 1-3% of children aged between 10 and 18 years [1,2]. AIS, characterized by lateral curvature of the spine and often accompanied by vertebral rotation, poses a physical challenge and significant psychological and social burden on affected individuals [3,4]. The condition emerges during the critical growth years, typically between the ages of 10 and 18, making early intervention vital to prevent severe deformities and associated complications in adulthood [5,6].

Traditionally, the gold standard for AIS treatment has been spinal fusion surgery to stop the progression of the curvature [7,8]. Over the years, pedicle screw constructs have gained prominence as essential tools in spinal fusion procedures [9]. These interventions provide enhanced stability and biomechanical support, facilitating better deformity correction. However, the optimal density of pedicle screw placement remains a subject of ongoing debate within the orthopedic community [10,11].

Recently, there has been a shift toward utilizing low-density pedicle screw constructs. Low-density constructs, while reducing the number of screws used, aim to achieve effective correction, thereby minimizing the surgical invasiveness and potential complications [12].

AIS is not merely a cosmetic concern; it can severely impact a young individual’s quality of life. Besides the visible deformity, scoliosis often leads to back pain, respiratory issues, and psychological distress [13]. The societal impact is important as adolescents navigate their formative years while grappling with the physical challenges posed by the condition [14].

The pressing need for a less invasive, highly effective surgical intervention is evident. The quest for achieving optimal deformity correction while minimizing the surgical effect has led researchers and clinicians to reevaluate the role of pedicle screw constructs in AIS surgeries. Striking a delicate balance between correction efficacy and invasiveness is pivotal, directly impacting the patients’ long-term well-being [15,16].

Historically, AIS correction has presented several challenges. Achieving three-dimensional correction, especially in cases with complex curvature patterns, demands a nuanced surgical approach [17]. The surgery should not hinder spinal growth, especially in younger patients. Balancing the need for immediate correction with the patient’s long-term spinal health and functionality necessitates a comprehensive understanding of the biomechanical principles involved [18].

Recent advancements in surgical techniques and evolving implant designs have opened avenues for innovative approaches to AIS correction. Pedicle screws, anchors in spinal fusion surgeries, have undergone design and placement strategy refinements. Low-density constructs, strategically placed to achieve maximal correction, have emerged as a potential solution to address the complications of traditional high-density screw constructs and non-operative management approaches [19,20].

This prospective study aimed to unravel the potential of low-density pedicle screw constructs in revolutionizing AIS management. By meticulously analyzing the outcomes of AIS patients treated with these constructs, this study aims to shed light on their efficacy, safety, and impact on the quality of life of the affected adolescents. Comprehensive radiographic assessments and patient-reported outcomes will provide valuable insights into the long-term implications of this novel surgical approach.

## Materials and methods

Study design

This prospective study involved 20 treatment-naive patients diagnosed with AIS who underwent surgical intervention employing low-density posterior pedicle screw fixation. The study was conducted at the Neurosurgery Department, Faculty of Medicine, Al-Azhar University (New Damietta) from March 2021 to March 2023. The study protocol was approved by the Faculty of Medicine, Al-Azhar University Research Ethics Committee (approval number: 21-05-006). All included patients provided written informed consent before study initiation.

Study population

Inclusion criteria were defined according to the following: (1) patients between the ages of 10 and 20 years with AIS; (2) all Lenke types of AIS diagnosis; 3) patients with a main thoracic curve of more than 40° and less than 80°; (4) posterior spinal fusion with pedicle screw constructs; (5) absence of a thoracoplasty; and (6) at least one-year follow-up in radiographic and Scoliosis Research Society (SRS) 22 outcomes.

Exclusion criteria

We excluded those with (1) previous spine surgery; (2) use of hooks or wires; (3) pedicle subtraction osteotomy, vertebral column resection, or vertebral column de-cancellation techniques; (4) syndromic scoliosis; (5) neuromuscular scoliosis; and (6) secondary scoliosis.

Sample technique

A non-probability (convenient) sampling technique was employed. All eligible individuals were invited to participate, and informed consent was obtained until the desired sample size was attained. The sample size was calculated using the OpenEpi Tool (https://www.openepi.com/). With an expected major Cobb mean difference pre and postoperatively of 37 ± 12, 80% power, and 95% confidence interval, the required sample was four patients.

Preoperative methods and recordings

A comprehensive medical history was gathered, including pain assessment, neurological symptoms, deformity progression, and prior treatments. Patients also completed the SRS-22 outcome questionnaire. We used the Arabic version of the SRS-22 questionnaire, which was validated previously [21]. Physical examinations included vital signs, shoulder balance, rib hump, waist asymmetry, pelvic tilt, leg length, and skin conditions. Detailed neurological examinations, including deep tendon and abdominal reflexes, were conducted.

Investigations

Imaging studies included plain X-rays of the entire spine and lateral bending views. To evaluate the anatomy (e.g., pedicle size and deformity) and achieve good surgical planning (e.g., detection of the degree of rotation and length and direction of safe pedicle screw in each level), we used the multi-slice CT (160-slice Toshiba Aquilion, Japan) for the dorsal and lumbar spine. Four views were collected, including coronal, sagittal, axial, and three-dimensional reconstruction. Additionally, MRI scans (Philips Achieva 1.5 Tesla, the Netherlands) of the dorsal and lumbar spine were performed to exclude any secondary cause for scoliosis (e.g., tethered cord). Routine laboratory investigations (e.g., complete blood count, random blood glucose level, liver function tests, kidney function tests, hepatitis B and C markers, and ABO grouping), respiratory function tests, and echocardiography (EPIQ CVx, Philips, Netherlands) were conducted to ensure comprehensive preoperative assessment.

Operative technique

Under general anesthesia, patients were placed in a prone position. Surgical steps encompassed incisions over the deformity, muscle dissection, pedicle screw insertion, facetectomy, Ponte osteotomy, and additional release procedures, as required. Instrumentation involved precise rod placement and translation of vertebrae. Fusion levels were determined based on the Lenke classification, with upper and lower instrumented vertebrae selected accordingly. Shoulder balance was assessed both clinically and radiologically.

Intraoperative and postoperative assessment measures

Operative time, blood loss, and any intraoperative complications were meticulously recorded. Patients were encouraged to mobilize post-surgery and were discharged after a few days. Follow-up visits were scheduled at two weeks, one month, three months, and six months post-surgery. Comprehensive clinical and radiological evaluations were performed at each follow-up, and any postoperative complications were documented.

Methods of final assessment

Functional outcomes were assessed using the SRS-22 questionnaire in Arabic administered preoperatively and at subsequent follow-up visits.

Statistical analysis

Statistical analysis was conducted using SPSS version 22 (IBM Corp., Armonk, NY, USA). Categorical variables were presented as frequencies and percentages, while continuous variables were presented as mean and standard deviation (SD). The association between categorical variables was tested using the chi-square test. The independent t-test or Mann-Whitney test was used to assess the association between continuous variables. The significance level was set at p-values <0.05.

## Results

Demographic and clinical characteristics

Our study included 20 patients, including 12 (60%) females, undergoing surgery at a mean age of 15.4 ± 1.57 years, with a mean follow-up period of two months (range: 6-40 months). The patients were categorized into different Lenke types, with five (25%) having main thoracic curves, one (5%) double thoracic, nine (45%) double major, one (5%) triple major, and three (15%) thoracolumbar/lumbar, while one (5%) had thoracolumbar/lumbar curves in conjunction with main thoracic involvement. In total, 13 (65%) had normal thoracic kyphosis (indicated by sagittal modifier n), while two (10%) showed reduced kyphosis (sagittal modifier -), and five (25%) exhibited increased kyphosis (sagittal modifier +), as shown in Table 1.

**Table 1:** Demographic data.

Variable		N (%)
Gender	Male	8 (40%)
	Female	12 (60%)
Age at surgery, years	Mean (SD)	15.4 ± 1.57
Follow-up, months	Mean (range)	12 (6-40)

Surgical data

The mean operative time was 219 ± 29.7 minutes, ranging between 180 and 310 minutes. The mean operative blood loss was 1,100 ± 250 cc, ranging between 400 and 1,900 cc, and 2 ± 1.5 units of blood were transfused. On average, each patient underwent fusion across approximately 11.8 ± 3.5 spinal levels and had an insertion of 16.5 ± 4.8 screws. The mean implant density was 1.4 ± 0.34, ranging between 1.3 and 1.45, as shown in Table 2.

**Table 2:** Surgical data.

Variables	Mean	Range
Operative time, minutes	219 ± 29.7	180-310
Operative blood loss, cc	1,100 ± 250	400-1,900
Operative blood transfusion units	2 ± 1.5	1-4
Number of fusion levels per patient	11.8 ± 3.5	7-17
Number of screws per patient	16.5±4.8	11-24
Implant density	1.4±0.34	1.3 - 1.45

Postoperative outcomes

Coronal and Sagittal Parameters

Regarding coronal parameters, the main curve Cobb angle reduced significantly from 61.33 degrees pre-surgery to 12.78 degrees post-surgery (p < 0.001). It reached 13.51 degrees at the final visit (p = 0.689), signifying a 79.14% correction overall. Similarly, the minor curve Cobb angle decreased substantially from 38.16 degrees pre-surgery to 11.87 degrees post-surgery (p < 0.001) and reached 12.86 degrees at the final visit (p = 0.546), demonstrating a noteworthy 68.91% correction. The coronal balance improved from 2.26 cm pre-surgery to 1.34 cm post-surgery (p = 0.003) and further enhanced to 1.05 cm at the final evaluation (p = 0.112). In sagittal parameters, thoracic kyphosis decreased significantly from 38.44 degrees pre-surgery to 29.76 degrees post-surgery (p < 0.001) and maintained at 30.36 degrees during the final visit (p = 0.264). Lumbar lordosis reduced from 47.62 degrees pre-surgery to 40.09 degrees post-surgery (p < 0.001) and further stabilized at 41.2 degrees in the final assessment (p = 0.135). The global sagittal balance improved from -8.5 mm pre-surgery to +3.7 mm post-surgery (p = 0.032) and remained stable at +2.7 mm at the final visit (p = 0.608), as shown in Table 3.

**Table 3:** Coronal and sagittal parameters. P-value 1: The difference between pre and postoperative values. P-value 2: The difference between the final visit and postoperative values.

Coronal parameters	Pre	Post	P-value 1	Final visit	P-value 2
Main curve cobb	61.33 ± 15.4°	12.78 ± 8.84°	<0.001**	13.51 ± 9.35°	0.689
Minor curve cobb	38.16 ± 15.4°	11.87 ± 11.09°	<0.001**	12.86 ± 9.62°	0.546
Coronal	2.26 ±1.46 cm	1.34 ± 1.44 cm	0.003**	1.05 ± 0.99 cm	0.112
Sagittal parameters	Pre	Post	P-value 1	Final visit	P-value 2
Thoracic kyphosis	38.44 ± 16.71°	29.76 ± 11.05°	<0.001**	30.36 ± 11.03°	0.264
Lumbar lordosis	47.62 ± 14.64°	40.09 ± 12.1°	<0.001**	41.2 ± 10.62°	0.135
Global sagittal balance	-8.5 ± 27.8 mm	+3.7 ± 27.5 mm	0.032*	+2.7 ± 23.1 mm	0.608

Complications

Complications were reported in four cases, each occurring once, including infection, adding-on phenomenon, proximal junctional kyphosis, and thoracic hypokyphosis.

Quality of Life

The findings from the SRS-22 questionnaire showcased notable improvements in the patient’s well-being following surgery. Pain scores increased from 3.68 to 4.22. Self-image ratings increased from 2.62 to 3.84, reflecting improved body confidence. Function scores remained relatively stable, slightly increasing from 3.43 to 3.54. Mental health ratings improved from 3.12 to 3.72, and satisfaction levels substantially increased from 2.2 to 4.28. The total score demonstrated a noteworthy improvement, increasing from 2.77 to 3.68, as shown in Table 4.

**Table 4:** Results of the Scoliosis Research Society (SRS) 22 questionnaire.

Domain	Preoperative	Last visit	P-value
Pain	3.68	4.22	<0.001
Self-image	2.62	3.84	
Function	3.43	3.54	
Mental health	3.12	3.72	
Satisfaction	2.2	4.28	
Total score	2.77	3.68	

## Discussion

This study investigated the efficacy of low-density posterior pedicle screw fixation in correcting AIS. Our findings demonstrated significant improvements in both coronal and sagittal parameters.

Although our study did not specifically focus on implant density, it demonstrated substantial deformity correction in AIS patients, significantly reducing both major and minor curve Cobb angles. Shen et al. explored low and high-density pedicle screw instrumentation and found similar clinical and radiological outcomes in Lenke 1 AIS patients, emphasizing the potential of both low and high-density constructs [22]. Tannous et al. showcased that a low-density screw construct achieved comparable curve correction to a high-density construct in adolescent scoliosis, emphasizing the effectiveness of low-density constructs [23]. Skalak et al. studied Lenke 2 AIS patients and found that increased implant density did not predict postoperative curve magnitude, highlighting the complexity of the relationship between implant density and correction [24]. Chotigavanichaya et al. compared different screw density patterns and found that very low-density and low-density constructs achieved similar radiographic correction while reducing operative time and cost, emphasizing the balance between correction and efficiency [25].

We demonstrated significant improvements in various domains of the SRS-22 questionnaire, reflecting enhanced quality of life and increased patient satisfaction post-surgery. Bharucha et al. emphasized the importance of clinical and radiographic outcomes in treatment decisions, aligning with our study’s focus on quality of life improvements as a vital parameter [26].

While our study did not explicitly analyze costs, it indirectly highlighted the importance of cost-effectiveness in surgical interventions. Bharucha et al. underlined the significant cost difference between high-density and low-density thoracic pedicle screw constructs, emphasizing the economic implications of implant choices [26].

We reported some complications, including infection and proximal junctional kyphosis, aligning with acknowledging potential post-surgical challenges.

The combined findings from our study and existing research underscore essential clinical considerations for managing AIS. Individualized treatment plans, considering patient-specific factors and financial considerations, are paramount. Prioritizing enhancements in the patient’s quality of life and satisfaction becomes pivotal, guiding healthcare professionals to focus on physical results and emotional well-being post-surgery [27]. Surgeons should balance implant density and efficiency, ensuring effective correction of deformities while optimizing costs [28]. Additionally, ongoing vigilance for potential complications such as infections and proximal junctional kyphosis is crucial, emphasizing the significance of post-surgical care [29]. Other studies reported neurological complications such as sensory impairment, postural headache, and thoracic paresthesia [30].

In our study, several strengths increased its credibility. Our research encompassed a diverse sample of 20 AIS patients, providing a comprehensive view of varied cases. Assessing clinical and radiological parameters and utilizing validated tools such as the SRS-22 questionnaire ensured a holistic evaluation of post-surgical outcomes. Additionally, our study explored aspects of deformity correction, implant density, and patient quality of life, contributing valuable insights to the existing body of knowledge.

However, certain limitations should be acknowledged. The relatively small sample size might limit the generalizability of our findings to a broader population. Furthermore, the short follow-up period, averaging two months, might not capture the long-term effects and complications that could arise post-surgery. Additionally, the absence of a detailed cost analysis in our study restricts a comprehensive understanding of the economic implications of different surgical approaches.

## Conclusions

Our study demonstrates the effectiveness of low-density posterior pedicle screw fixation in correcting AIS, leading to improvements in both coronal and sagittal parameters. These positive outcomes have significant implications for the clinical management of AIS patients. We recommend further research with larger sample sizes and longer follow-up durations to confirm these corrections’ durability and impact on patients’ long-term quality of life. Surgeons should consider this technique in appropriate cases, ensuring careful patient selection and thorough preoperative assessment to optimize surgical outcomes.
